# Social network sites as learning environments and their implications for mental health

**DOI:** 10.3389/fdgth.2022.939740

**Published:** 2022-10-10

**Authors:** Felix S. Hussenoeder

**Affiliations:** Institute of Social Medicine, Occupational Health and Public Health, Leipzig University, Leipzig, Germany

**Keywords:** social network sites, learning from feedback, learning from observation, digital environments, mental health, theory, technology

## Abstract

Social network sites (SNSs) have become ubiquitous around the globe and interwoven with all aspects of life. In this article, I will argue that the communicative infrastructure of SNSs, i.e., all SNS-elements that allow users to communicate, is a key element for understanding their impact as it creates environments in which users, their behaviors, and social interactions are embedded. These digital environments facilitate and encourage fundamental mechanisms of implicit learning from feedback as well as observation in an unprecedented way. I will discuss how these technology-based learning environments impact the mental health of their users, e.g., by linking negative online feedback to depression and following influencers to disturbed eating. The article ends with a conclusion that emphasizes the advantages of understanding SNSs as environments in order to reflect the complexity, relevance, and ubiquitousness of the phenomenon.

## Introduction

Social network sites (SNSs) have become ubiquitous in the everyday lives of people around the globe. They have become places for social connection, interaction, and communication as well as for entertainment, information seeking, self-expression, and commerce [e.g. (1–3)], and they have managed to blend with the world of social relations, businesses, and political parties to a degree that the traditional separation between offline and online has become obsolete.

On a very general level, SNSs are “bundles of technological tools that incorporate features of earlier technologies (such as personal websites) but recombine them into a new context that supports users’ ability to form and maintain a wide network of social connections” (4). Besides these fundamental commonalities, SNSs exhibit multiple differences in terms of popularity, target group (e.g., business people, photo enthusiasts, researchers, everyone), functions (connecting with friends, career, dating), costs and access restrictions, design, and specific features (5). The focus of this article is on the most popular and rather unspecific SNSs where a great variety of users connects with a wide array of contacts, from family to strangers, to pursue a diverse set of (online) activities like commenting, sharing pictures or engaging in discussions. Typical examples are Facebook, Instagram, WeChat, or VK. However, the mechanisms discussed in this article can to some extent also be found with other SNSs and even other forms of social media like media sharing networks or discussion forums.

SNSs have become a meta-context that is woven into our everyday reality, from living rooms and birthday parties to schools, businesses, and cafés. Most likely there is already someone sharing your party pictures while you are celebrating, or rating the café latte you are about to have. With their pervasiveness, inclusiveness, and permanence, SNSs represent digital environments that are ubiquitous yet elusive. Scholars have tried to capture digital environments by referring to their *architecture* as a metaphor for the “composite result of structure, design and organization” of SNSs (6) and “the technical protocols that enable, constrain, and shape user behavior in a virtual space” (7). They have showed that digital architecture can affect norms of interaction (Papacharissi) as well as political communication (Bossetta).

While *architecture* is intuitive, it is also highly unspecific and does not differentiate between different aspects of the environment. In this article, I want to go one step further by focusing on a key element of SNS architecture: their communicative infrastructure (CI), i.e., all SNS-elements that allow users to communicate, including personal profiles, instant messaging, and groups. The focus on CI—rather than on architecture as a whole—can help scholars to avoid conceptual ambiguity, and to better understand the practical implications of SNS, e.g., in terms of mental health of users. While there are minor differences between SNSs, most CIs share three fundamental properties. First, the CI connects users to large digital networks of contacts. Since those contacts are associated with profile information, digital networks are information-rich, which makes them qualitatively different from offline networks of acquaintances. Second, the CI can be used to communicate with anyone in the network (and to some extent beyond), at any time, from any place as long as there is a device and an internet connection. In addition, there are very few barriers to the initiation of communication, e.g., in the form of office hours, lack of contact data, or required effort. Third, most online communication is visible to a large number of contacts, over a long period of time, and contacts could easily engage in it.

The CI is at the same time omnipresent and invisible. It represents the environment in which SNS users, their behaviors, and social interactions are embedded, and it links users to their networks. It therefore shapes every activity that takes place on SNSs and beyond, from self-presentation and social interaction to collective action. The CI affects users by encouraging, facilitating, and supporting learning *via* feedback and observation. While it is highly plausible that the CI affects users in a variety of other areas, from career building to dating, the focus of this article is on mental health implications.

## The CI as an implicit learning environment and its implications for the mental health of users

While the term “learning” often evokes pictures of classrooms, teachers, and exams, its meaning is much more broad, fundamental, and encompassing. Learning can take place in any environment, at any time, and it can refer to almost everything, from riding a bike, baking bread, and behaving in a socially acceptable way to quantum physics, a language, and dealing with pain. In a very broad sense, learning is the “acquisition of knowledge or skills through study, experience, or being taught” (e.g., lexico.com), and there is a general agreement that it involves a relatively permanent change in behavior due to past experience (8). However, since learning is such an important concept in so many areas, there is also a great variety of definitions and approaches from disciplines like psychology, evolutionary theory, and computer science (9). In addition, learning does not have to be beneficial as learning processes are involved in addiction, antisocial behaviors, and violence (10–12).

Learning can be differentiated in explicit and implicit forms. Hulstijn defines explicit learning as “input processing with the conscious intention to find out whether the input information contains regularities and, if so, to work out the concepts and rules with which these regularities can be captured” and implicit learning as “input processing without such an intention, taking place unconsciously” [(13), p. 131]. In this article, the focus is on the CI as an implicit learning environment since on SNSs the majority of learning takes place implicitly, as an unintended side product of other SNS activities.

Learning requires input, and on SNSs input will take the form of information, e.g., a text, picture, link, or video. Therefore, the question is how one specific piece of information—out of the abundance of information on SNSs—becomes effective input in terms of learning. We can formulate some highly plausible theoretical preconditions:

(1) *Availability*. The information has to be available in the CI at a specific time and space. This also means that the information has to be in digital form.
(2) *Perception*. The user has to encounter the information, and they have to be able to perceive it. The capability to perceive information is affected by factors inside the user, like cognition, motivation, and attention, as well as external factors, like distractions in the physical environment. In addition, the design and structure of the digital environment can facilitate or hinder the perception of information, e.g., *via* short vs. long presentation times, repeated, targeted, contextualized, emphasized, or marginalized presentation, the use of pictures and colors, or reduced font size. Once the user has perceived a piece of information, its status changes from noise to input in terms of learning. Due to the large amount of information in the CI, most information perception takes place on a subconscious, implicit level. While availability is the precondition for perception, it is nevertheless not sufficient.
(3) *Relatedness*. Information is more likely to become input when there is a relationship between information and user, e.g., with their personality, attitudes, motivations, goals, interests, values. Information can become input by supporting one's opinion, inducing positive feelings, building confidence, satisfying curiosity, and boosting self-esteem, but also by threatening beliefs, attacking values, or disrespecting attitudes.

Users are receivers of input but they also shape the learning environments of other users by providing, sharing, and evaluating information. However, the impact of regular, individual users on the learning environments of others is rather small and inconstant due to large network sizes.

There is a plethora of learning theories and concepts in the literature [e.g., see (14)], and approaches like *connectivism* that emphasizes the crucial role of large, diverse, and (technology based) networks for learning and knowledge are reflective of the digital reality of SNSs (15, 16). However, the focus of this article is another one. It is on two fundamental, pervasive, and well-established learning mechanisms which are hardwired, not only into the human brain but also into the digital environments. These mechanisms are linked to feedback and observation, and they can affect the mental health of users. As Valkenburg et al. (17) argued in their review with regard to inconsistent research findings, social media use can have different effects on different users, and negative mental health outcomes may specifically affect vulnerable subgroups of users.

### Learning from feedback

One of the most basic and intuitive learning mechanisms is the modification of behavior *via* feedback (18), i.e., animals and human beings are more likely to exhibit behaviors for which they get rewarded, and they will reduce or abandon those that they get punished for (19). Research on feedback learning belongs to the foundations of modern psychology, from the proverbial Pavlovian dog to the highly influential work of the American behaviorists Skinner, Watson, and Thorndike (20–23). Since the CI promotes the visibility of communication, almost every act of online communication, from making a contact to sharing a post and commenting, is a behavior performed in front of an audience, similar to what sociologist E. Goffman described as front stage performance (as opposed to back stage) in his famous theatrical metaphor (24).

Because the SNS-audience is technologically empowered and encouraged to interact, it is very likely that it will react and provide feedback which is either rewarding, e.g., positive, affirming or encouraging comments, Facebook likes, and re-sharing, or punishing, e.g., negative, derogatory, and insulting comments or little/ no feedback. While in most digital interactions users do not explicitly ask for feedback, some users actively utilize social media to receive feedback and validation (25, 26).

The CI does not only support and encourage mechanisms of feedback giving between SNS users, it also has the potential to promote the effectiveness of feedback. The fact that the CI stimulates and accumulates feedback in an unprecedented way could facilitate the effects of CI-based feedback as some studies suggest that the effects of feedback on perceived helpfulness, student performance, manager effectiveness, and online behaviors increase with the amount of feedback given (27–30). In addition, due to the pervasive, instantaneous, and highly connective nature of the CI, feedback is likely to be immediate. Feedback immediateness is an additional factor that supports learning, especially when it takes place on an implicit, automatic level that requires little cognitive elaboration (31–33). Moreover, the continuous availability of feedback information that is a core element of most SNSs was linked to successful behavioral learning in intervention studies (34).

#### Implications for mental health

Feedback mechanisms play a crucial role in mental health and in the formation and maintenance of mental disorders. For example, research showed associations between feedback-seeking and depression (35), biased interpretation of social information and anorexia nervosa (36), and a focus on negative feedback and borderline personality disorder (37). With the high relevance of feedback for mental health, it is not surprising that there are multiple studies connecting positive as well as negative feedback on SNSs to mental health outcomes.

The permanent availability of instant positive feedback is unparalleled in the offline world, making SNS use a highly rewarding experience that can be accessed by anyone, at any time, and with little effort. As a consequence of its rewarding nature and its permanent availability, SNS use can easily become addictive. Feedback learning plays a crucial role in SNS addiction as studies showed that receiving (and even giving) positive feedback on SNSs coincides with the activation of cerebral reward structures (38, 39) and an increase in self-esteem (40). Addictive SNS use has been linked to reduced academic and job performance (41, 42), poor sleep quality (43), decreased satisfaction with life and wellbeing (44, 45) and increased anxiety (46).

On the opposite side, online feedback can also be negative, derogatory or threatening. Studies suggested an empirical connection between negative feedback on Facebook and disordered eating attitudes (47) as well as between getting fewer online likes than others and the experience of rejection, negative affect and thoughts, and more depressive symptoms (48). A recent review showed that cyber harassment, i.e., an extreme form of negative feedback online, is associated with a wide range of mental health problems including depression, anxiety, stress, and anger (49).

Feedback mechanisms on SNSs affect different users in different ways, and users that engage in risky online self-presentation seem to be at a higher risk for receiving negative feedback (50). Seeking feedback on SNSs has been linked to reduced self-esteem (51) and depressive symptoms, especially when self-worth depends on online feedback (26, 52). In addition, a current study suggested that the higher rates of depressive symptoms in LGBTQ persons could to some extent be explained by their exposure to negative experiences on social media including being called out or hurt, receiving negative or no feedback, and being excluded (53).

### Observational learning

Observational learning “is concerned with the acquisition of attitudes, values, and styles of thinking and behaving through observation of the examples provided by others” (54). There is an extensive literature over a long period of time showing that observational learning is a powerful learning mechanism for a wide range of behaviors from food choice (55) to environmentally responsible (56) as well as violent behaviors (57). In the context of socialization processes, observational learning shapes values, norms, and identities (58–60).

On SNSs any act of communication is a potential learning example. This is obvious when users explicitly state their interests, values and preferences or share their opinions, but it goes far beyond that. For example, from a snapshot at a graduation party viewers could learn about the correct dress code, cultural norms and values in the context of celebrating achievements, table manners, and personal taste. By sharing vegan cooking recipes, posting music from nationalist bands, or sharing pictures from LGBTQ events, users act as role models and they implicitly (and sometimes explicitly) convey attitudes, values, and norms.

In addition to traditional facilitators of observational learning like friends and family members, SNS users are encouraged to “follow” stars and celebrities, mainly from entertainment and sports, which affect their audiences in multiple areas. Furthermore, SNSs have become the breeding ground for their own celebrities: influencers that accumulate a relatively large following through the narration of their personal lives and lifestyles, who engage with and monetize their following (61). Being ordinary social media users themselves (as opposed to traditional celebrities), influencers can even more relate to the lives and experiences of SNS users and therefore become ideal role models, even though their influencing rarely transcends the narrow realm that is defined by commercial interest (62). In addition, research unrelated to SNSs connected celebrity endorsement to buying behavior (63), female sports role models to gender equity and empowerment (64), and celebrity suicide to changes in suicide rates (65).

Users are not left alone with evaluating the acceptability, likability, inappropriateness, or hideousness of digital content, they can rely on the co-evaluation done by the contacts in their networks *via* comments, likes, or shares. In that way the previously discussed abundance of feedback and its meaningful accumulation, e.g., in the form of likes, function as an easily accessible and intuitive indicator for the relevance and meaning of content.

#### Implications for mental health

Observational learning plays a crucial role in mental health. For example, it has been connected to pain (66, 67), anxiety and fear (68, 69), drug dependence (10, 70), and eating disorders and obesity (71, 72). The studies above cover a wide range of contexts and situations from infants learning from their mothers over peer-influence to role models on TV, emphasizing the crucial role of observational learning in mental health.

By joining an SNS, users receive a permanent stream of posts, news, pictures etc. related to the lives of their contacts. Because the CI is supporting the generation of content and the connection with others, SNSs are full of observable content. In addition, since users have control over what they share in an online setting more than they would have offline, the information displayed on SNSs is mainly positive, e.g., related to joyful events and activities, individual achievements, or coveted material possessions (5). Due to this simultaneously realistic and positively biased self-presentation, learning from observation can easily result in detrimental comparisons. Studies connected social comparisons online to lowered wellbeing and self-esteem, self-injury, and suicidal behavior (73–75), and two recent meta-analyses linked online social comparisons to reduced wellbeing and depression (76, 77). Long-term and frequent users seem to be more likely to be affected by these comparisons (78).

While the previous studies connected social media use to a variety of mental health problems, SNSs seem to be especially detrimental when it comes to eating as studies showed associations between social media use and concerns around eating and body image, disordered eating, and body change behaviors (79–81).

When it comes to eating-related health outcomes, social comparisons on SNSs play a major role as studies connected them to disordered eating (82), and exposure to image-related content to increased body dissatisfaction, food restriction, and overeating (83). These associations can be understood as the consequences of a CI-based learning process. I.e., on SNSs users learn about the fitness routines, eating habits, and personal success stories of others from a permanent stream of statements, notifications, and pictures, and as a consequence they experience and evaluate their own habits, bodies, and achievements in the context of that information. Since SNSs are biased towards the sharing of positive content (5), this experience can easily become detrimental and disappointing for users. It is therefore no surprise that compared to in-person comparisons, upward comparisons on SNSs were connected to a less favorable body image and more negative mood (84).

The fact that disordered eating behaviors show such a strong relationship with SNSs can be seen as a consequence of the cultural and social context in which SNS use takes place that puts a high value on physical attractiveness and thinness. Thoughts, ideas, and behaviors that are associated with disordered eating—like excessive food restriction and dieting, obsessive concern with nutrition, and body dissatisfaction—are much more in line with the cultural setting and the zeitgeist than those associated with other mental health issues. Hence, they are more likely to be displayed, observed, and further internalized. This dynamic increases with the amount of comparable information in the CI which is reflected in the empirical connection between network size and appearance-related pressures (85). In its most extreme form the close relationship between social media use and disordered eating is reflected in pro-anorexia groups on SNSs that celebrate anorectic behaviors as a lifestyle choice and reject the classification of anorexia as an illness (86, 87). In these cases, processes of observational learning are intensified by an increased exposition to anorectic content.

While there is little research on the mental health effects of social media influencers on their followers, studies already connected following influencers to envy (88), consumption of unhealthy beverages in children (89), and perceptions of inferiority in individuals who are socially anxious (90).

By providing an abundance of potential examples and an environment of permanent ubiquitous feedback, the CI facilitates implicit learning. In addition, the wealth of information on SNSs that I discussed in the context of feedback and observational learning is unprecedented in human evolution, and studies showed that the resulting social media overload can be linked to stress (91), reduced wellbeing (92) and increased distress (93).

## Conclusion

In this article, I introduced the concept of digital learning environments to better understand the effects that SNSs have in the daily lives of billions of people around the globe. The environment perspective has multiple advantages as it emphasizes the complexity of the phenomenon, the embeddedness of users, their behaviors, and interactions, and the ubiquitousness of the technology. It also highlights the fact that there is a large and diverse number of potential input factors that can affect users, and it shifts focus to the processes and mechanisms that underlie environmental impact.

I discussed the nature, relevance, and impact of digital learning environments with regard to mental health, an impact that will change and grow in the future. Already over the last two decades SNSs have developed from a niche product to a global phenomenon and their evolution will continue further. Recently, this process has been fueled by the widespread use of SNSs on mobile devises and the rise of new social media like TikTok. In addition, the ongoing extension of the digital world and the amalgamation of the digital with the non-digital realm, e.g., with regard to augmented and virtual reality and the internet of things, will shape the future of social media and probably create even denser, more encompassing, and more immersive digital environments. Hence, SNSs will continue to play a crucial role in the lives of their users as well as in shaping the social world.

Digital environments are by no means always deterministic, problematic, and detrimental, and recent research showed that SNS use can have positive effects on mental health and wellbeing (94, 95), and that observational learning online could increase political participation (96). Nevertheless, it is important to realize that the CI plays an active role, it is literally creating worlds and causing severe repercussions for users. It is therefore important that SNSs and the technology they impose on their users become more transparent, and that users can understand, build, and control their digital environments. Hence, regulators need to address the extreme imbalance of power between SNS owners and users when it comes to designing and shaping digital environments. Last but not least, future SNSs should be build around the interests and needs of users rather than those of platform owners. It is time to take care of the environment!

## Data Availability

The original contributions presented in the study are included in the article/Supplementary Material, further inquiries can be directed to the corresponding author/s.
